# Impact of Acute Hex-Bar Deadlift Exercise on Subsequent Cycling Performance and Skeletal Muscle Oxygenation

**DOI:** 10.3390/sports14100437

**Published:** 2026-10-07

**Authors:** Arturo Sosa, Larissa M. Barreto, Julieta R. Sanchez, Ivan J. Leon, Monica Alcantara, Emily Percino, Jennifer T. Lin, Mekhii R. Morris-Heron, Carlos A. Estrada, Michael D. Belbis

**Affiliations:** 1 Department of Kinesiology and Physical Education, Northern Illinois University, DeKalb, IL 60115, USA; z1919948@students.niu.edu (A.S.III);; 2 Department of Exercise Science, Aurora University, Aurora, IL 60506, USA

**Keywords:** resistance exercise, post-activation performance enhancement, skeletal muscle oxygenation, near-infrared spectroscopy, cycling, oxygen uptake kinetics, exercise tolerance

## Abstract

Post-activation performance enhancement (PAPE) strategies are used to improve power-dependent performance, but their impact on endurance exercise and skeletal muscle oxygenation is less clear. We tested the hypothesis that acute hex-bar deadlift (HBD) exercise would improve cycling performance and vastus lateralis muscle oxygen saturation (S_m_O_2_). Twenty-five healthy young adults (15 men, 10 women) completed moderate- and severe-intensity cycling after HBD or dynamic stretching (DYN) in a randomized crossover design. HBD consisted of 3 × 5 repetitions at 70–80% of one-repetition maximum, followed by ~8 min of recovery before cycling. Severe-intensity time-to-exhaustion was the primary outcome, while heart rate, arterial blood pressure, pulmonary oxygen uptake (V̇O_2_), and S_m_O_2_ were assessed as secondary physiological outcomes. Time-to-exhaustion was not different between HBD and DYN (394 ± 36 vs. 381 ± 34 s; *p* = 0.692). Resting and end-contraction V̇O_2_ and S_m_O_2_ were not different between conditions during moderate- or severe-intensity cycling. Similarly, S_m_O_2_ kinetics and area under the S_m_O_2_ curve remained unchanged. No condition-related differences in V̇O_2_ kinetics remained significant after correction for multiple comparisons. Contrary to our hypothesis, acute HBD exercise did not enhance vastus lateralis muscle oxygenation or severe-intensity cycling tolerance in healthy adults.

## 1. Introduction

Skeletal muscle microvascular oxygenation is determined by the dynamic matching between local oxygen (O_2_) delivery and utilization [1,2,3,4]. Due to limited and finite O_2_ stores in the human body, the transition from rest to exercise requires rapid, coordinated adjustments across the O_2_ transport pathway, including increases in pulmonary gas exchange, cardiac output, exercising limb blood flow, microvascular red blood cell flux, and mitochondrial O_2_ utilization [2,4,5]. When these responses are not adequately matched to the metabolic demands of muscle contraction, skeletal muscle oxygenation decreases, the O_2_ deficit increases, and exercise tolerance may be compromised [5,6]. Thus, interventions that acutely modify neuromuscular activation, metabolic demand, or local O_2_ transport provide a useful experimental approach to examine the physiological determinants of endurance exercise performance.

Post-activation performance enhancement (PAPE) refers to an acute improvement in voluntary performance following high-intensity voluntary conditioning contractions [7,8]. Although PAPE is often discussed in parallel with post-activation potentiation (PAP), the terms are not synonymous. PAP refers specifically to enhanced electrically evoked twitch force, whereas PAPE describes improved voluntary performance without necessarily confirming classical twitch potentiation [7,8]. The mechanisms underlying PAPE are likely multifactorial and may include increased muscle temperature, altered muscle water content, enhanced motor unit recruitment, changes in muscle activation, and a favorable balance between potentiation and fatigue [7]. From an applied standpoint, these responses have led strength and conditioning professionals to use interventions, such as heavy squats, sled pulls, plyometric exercise, and deadlift variations, as acute conditioning activities before subsequent exercise performance tasks.

Most of the literature on PAPE has focused on explosive or power-dependent outcomes. Meta-analytic evidence supports the ability of conditioning contractions to improve jump, sprint, throw, and other ballistic performance, although the magnitude and direction of the response depend on various factors, such as the conditioning activity, load, volume, recovery duration, training status, and the individual’s strength level [9,10]. In contrast, the impact of PAPE on endurance exercise performance is less defined [11,12]. This distinction is important because endurance performance is not governed solely by peak muscle force production or neuromuscular power. Rather, exercise tolerance during cycling also reflects the integrated regulation of central and peripheral O_2_ transport, oxidative metabolism, metabolite accumulation, neuromuscular fatigue, and perceptual strain [5,13,14,15,16,17]. Therefore, a conditioning activity that improves force-generating capacity may not necessarily improve cycling performance if residual fatigue or metabolic perturbation offsets any potentiating effect.

There is, however, a strong physiological rationale for examining whether acute resistance exercise can alter subsequent cycling performance. Prior heavy exercise, often referred to as “priming” exercise, can alter pulmonary oxygen uptake (V̇O_2_) kinetics, reduce the V̇O_2_ slow component, and improve severe-intensity exercise tolerance under some conditions [18,19,20,21,22]. In skeletal muscle, prior contractions may also influence the subsequent microvascular O_2_ response by altering the balance between O_2_ delivery and O_2_ utilization at the onset of exercise [23]. Accordingly, any ergogenic effect of a PAPE stimulus during cycling may not be restricted to neuromuscular potentiation. Acute resistance exercise could increase muscle temperature, local vasodilatory tone, and blood perfusion, thereby improving the matching of O_2_ delivery to O_2_ utilization during subsequent contractions [4,7,23,24,25,26]. However, if the same stimulus induces residual fatigue, metabolic strain, or a greater O_2_ cost of exercise, these potential benefits may be offset [17,27,28,29,30].

Near-infrared spectroscopy (NIRS) provides a non-invasive approach to evaluate skeletal muscle oxygenation during dynamic exercise [6,31]. Although NIRS-derived muscle oxygen saturation does not directly measure blood flow, it reflects the local balance between microvascular O_2_ delivery and muscle O_2_ utilization within the interrogated tissue volume [6,31,32]. This makes NIRS particularly useful when evaluating interventions that may alter both perfusive and metabolic components of the O_2_ transport pathway. In the context of PAPE, an increase in skeletal muscle oxygenation during subsequent cycling would suggest that the conditioning activity improved local O_2_ delivery relative to utilization. Alternatively, unchanged or reduced oxygenation would suggest that acute resistance exercise did not meaningfully improve peripheral O_2_ delivery, or that any potential benefit was counterbalanced by greater O_2_ utilization or fatigue.

Despite the extensive literature examining PAPE and anaerobic performance, whether acute resistance exercise alters skeletal muscle oxygenation during subsequent endurance exercise remains unclear. This represents an important gap because previous studies evaluating PAPE strategies in cycling have reported mixed findings, with some demonstrating improved cycling performance following prior heavy strength exercise, high-intensity resistance-based warm-up, or drop jumps [33,34,35], while a recent meta-analysis does not support PAPE as a reliable ergogenic strategy for endurance exercise performance [11]. Recent work in endurance-trained individuals further suggests that PAPE responses may vary according to athlete-specific characteristics, reinforcing the need to examine both performance and physiological responses under specific experimental conditions [36]. Importantly, these studies have largely emphasized performance outcomes, while comparatively less attention has been given to the central and peripheral physiological responses that may explain those outcomes. Therefore, simultaneous assessment of pulmonary V̇O_2_, heart rate, arterial blood pressure, skeletal muscle oxygenation, and time to exercise intolerance may help determine whether the effects of acute resistance exercise extend beyond neuromuscular performance and into the regulation of O_2_ transport during cycling.

The purpose of the present investigation was to determine the effects of acute hex-bar deadlift exercise, used as a conditioning activity intended to induce PAPE, on subsequent cycling performance and skeletal muscle microvascular oxygenation in healthy young adults. Participants completed moderate- and severe-intensity cycling following hex-bar deadlift or dynamic stretching conditions in a randomized crossover design, while pulmonary gas exchange, cardiovascular responses, quadriceps muscle oxygenation, and exercise tolerance were assessed. Moderate-intensity cycling was included to evaluate whether HBD altered V̇O_2_ and S_m_O_2_ responses during a fixed-duration submaximal exercise bout, whereas severe-intensity cycling was used to determine whether these responses were altered under greater metabolic stress and translated to greater exercise tolerance. Considering the above, we hypothesized that acute hex-bar deadlift exercise would increase skeletal muscle oxygenation during subsequent cycling and improve exercise tolerance during severe-intensity exercise.

## 2. Materials and Methods

Participants were recruited from Aurora University in Aurora, IL, USA. The recruited population consisted of healthy men and women aged 18 to 35 years. Exclusion criteria included cardiovascular, respiratory, and/or neuromuscular disease; orthopedic limitation to resistance exercise and/or cycling; hypertension (resting arterial blood pressure > 140/90 mmHg); smoking; pregnancy; type 1 or type 2 diabetes mellitus; and/or hepatic or renal disease. Participants were required to have at least 3 months of resistance training experience to ensure familiarity with the resistance exercise protocol. To avoid the confounding effect of baseline training status, individuals who participated in formal athletic competitions (e.g., collegiate athletes) or regularly followed supervised exercise programs were excluded from the study. Procedures were approved by Aurora University’s Institutional Review Board (Project No. 1215) and conformed to the most recent version of the *Declaration of Helsinki*. All participants provided written informed consent before enrollment.

### 2.1. Experimental Design

After 30 individuals were screened for eligibility, 26 met all inclusion criteria, and 25 participants completed the study. Prior to recruitment, the planned recruitment target was set at 30 participants based on the feasibility of the randomized crossover design and prior PAPE-related cycling/performance literature, with severe-intensity time-to-exhaustion designated as the primary performance outcome [9,33,34,35]. The original planning records did not retain the assumed raw HBD–DYN mean difference, variability estimate, or software output; therefore, the original recruitment target should be interpreted as a planned sample target rather than a fully reproducible formal power calculation. To provide quantitative context for the final analyzed sample, a sensitivity analysis was performed using a two-tailed paired-samples framework with α = 0.05 and power = 0.80. With *n* = 25, the smallest detectable standardized within-subject effect was Cohen’s *d_z_* = 0.58; the planned recruitment target of *n* = 30 would correspond to *d_z_* = 0.53. Since the sample-size rationale was based on the primary performance outcome rather than the full set of secondary physiological outcomes, including pulmonary V̇O_2_ kinetics, muscle S_m_O_2_ kinetics, HR, and arterial blood pressure, the present analyses of cardiorespiratory and skeletal muscle oxygenation responses should be considered exploratory. The final sample size reflects the number of participants who met eligibility criteria and completed all experimental visits. The individual who met the inclusion criteria but did not complete the study voluntarily chose to discontinue participation.

Following screening and enrollment, each participant reported to the laboratory on four different occasions within a 2–3-week period. Among participants included in the final analyses, the average intervals between Visits 1 and 2, Visits 2 and 3, and Visits 3 and 4 were (4.4 ± 0.3), (3.5 ± 0.3), and (4.4 ± 0.5) days, respectively. The interval between Visits 3 and 4 represented the washout between experimental conditions. Participants were instructed to avoid strenuous exercise in the 24 h preceding each test session, refrain from eating for 3 h prior to exercise tests, and refrain from caffeine and alcohol intake for at least 12 and 24 h before each test, respectively. Menstrual-cycle phase and hormonal contraceptive use were not recorded or controlled, and experimental visits were not scheduled according to menstrual-cycle phase. All experimental procedures were performed at approximately the same time of day (±2 h) for each participant.

**Figure 1:**
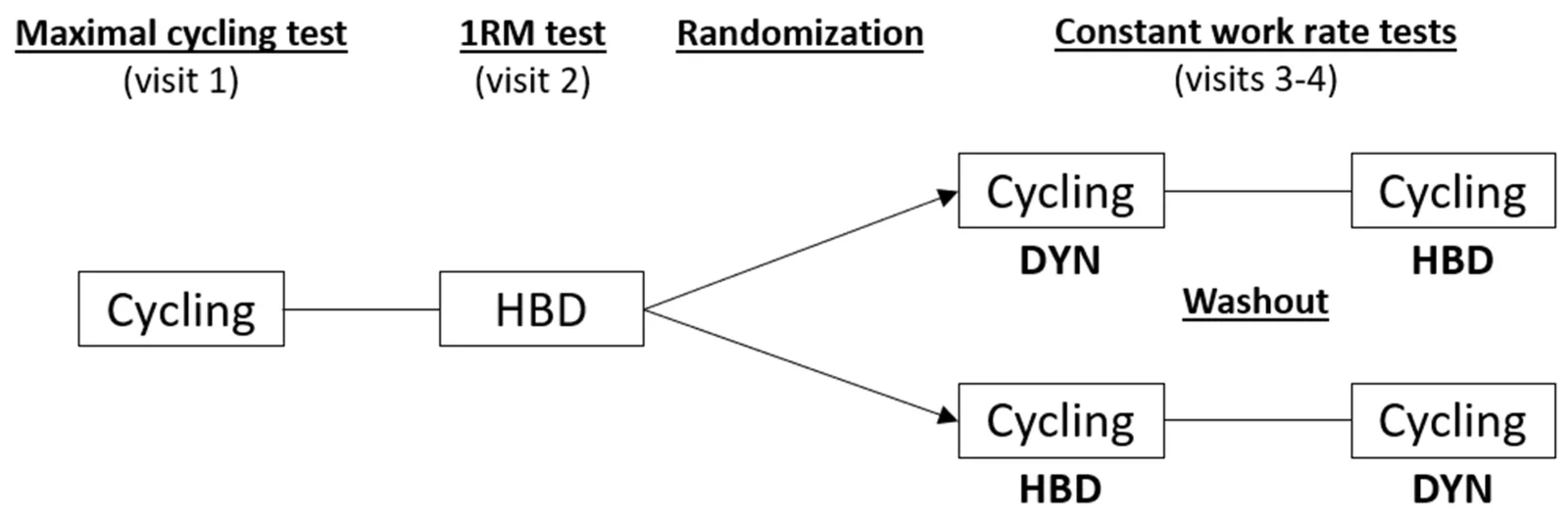
Flow diagram representing the study design. HBD, hex-bar deadlifts; DYN, dynamic stretching. Participant recruitment and completion details are provided in Section 2.1.

During visit 1, participants completed screening procedures and a maximal incremental cycling test to determine work rates to be employed during moderate- and severe-intensity cycling tests in subsequent visits (i.e., visits 3 and 4). During visit 2, participants completed a maximal strength assessment to determine one-repetition maximum (1RM) for the hex-bar deadlift (HBD). During visits 3 and 4, participants completed moderate- and severe-intensity cycling following either HBD exercise, used as the conditioning activity intended to induce PAPE, or a dynamic stretching control condition (DYN) as described below. Experimental conditions were completed in a randomized crossover design and separated by at least 3 days. Condition order was assigned using an online randomizer to determine whether participants completed DYN or HBD first. Allocation was not blocked, stratified, or formally counterbalanced; however, each participant completed both conditions. Randomization was not stratified by sex; therefore, sex balance across condition-sequence groups was not intentionally controlled. Given the exploratory nature of the study and the sample size, men and women were pooled for the primary analyses, and sex-specific or sex × condition-sequence effects were not evaluated. The experimental design is displayed in Figure 1.

### 2.2. General Exercise Procedures

All cycling tests were performed on a mechanical, pendulum-resistance cycle ergometer (Monark 828E Ergomedic; Monark Sports & Medical; Vansbro, Dalarna County, Sweden), at a pedal cadence of 70 rev/min. Pedal cadence was guided using a metronome, and participants were instructed to maintain the required cadence throughout all exercise tests. Seat height was recorded during visit 1 and replicated during subsequent cycling visits. Strong verbal encouragement was provided during maximal incremental and severe-intensity constant-work-rate tests. Exercise tests were terminated when participants signaled to stop pedaling or could not maintain the required pedal cadence for ≥5 s despite strong verbal encouragement.

### 2.3. Visit 1: Maximal Incremental Cycling Test

Upon arrival to the laboratory, participants completed a health and medical history questionnaire, followed by measurements of resting arterial blood pressure, height, and body weight. Participants were then fitted with a heart rate (HR) chest strap and metabolic mask before completing a dynamic warm-up. The dynamic warm-up included knee hugs into a lunge, quadriceps stretches, side lunges, straight-leg kicks, leg sweeps, side squats, high knees, and butt kicks.

Participants performed an incremental step test to determine the gas exchange threshold (GET), peak pulmonary oxygen uptake (V̇O_2peak_), peak work rate (WR_peak_), and work rates to be employed in subsequent visits (i.e., visits 3 and 4). Participants rested quietly on a chair for at least 5 min before transferring to the cycle ergometer. After 2 min of resting data collection on the cycle ergometer, participants began cycling at 17 W with an incremental step rate of 17 W/min (adjusted every min) until volitional exhaustion. HR was measured beat-by-beat using a sensor strapped to the thorax. Gas exchange data during the incremental test were averaged every 10 s. The GET was determined using the V-slope and ventilatory equivalent methods [37,38]. V̇O_2peak_ was taken as the highest 10-s average value attained before exercise termination. The work rates that would require 90% GET (moderate intensity) and 80% of WR_peak_ (severe intensity) were subsequently determined, taking into account the mean response time for V̇O_2_ during incremental exercise [39].

### 2.4. Visit 2: Hex-Bar Deadlift 1RM Testing

During visit 2, participants completed a maximal strength assessment to determine HBD 1RM using an Olympic High Hex Trap Bar (OBIS-88HB; CAP Barbell, Houston, TX, USA), which was also used during the HBD condition on experimental visits. Participants completed the same dynamic warm-up described above. Participants then performed a progressive HBD warm-up consisting of 5 repetitions at ~50% of the expected 1RM, 3 repetitions at ~70–75% of the expected 1RM, 2 repetitions at ~80–85% of the expected 1RM, and 1 repetition at ~90–95% of the expected 1RM. Participants rested 2 to 4 min between sets. After the final warm-up set, participants attempted a 1RM lift. If the lift was successful, resistance was increased, and another 1RM attempt was performed after 2–4 min of rest. This process continued until failure or until a maximum of 5 attempts had been completed. The heaviest successfully completed lift was recorded as the participant’s HBD 1RM. No separate familiarization visit was performed. However, participants received instruction and technique feedback during the dynamic warm-up and progressive HBD warm-up sets before maximal attempts. Proper lifting technique was visually assessed by trained research personnel, and only lifts completed with appropriate mechanics were accepted.

### 2.5. Visits 3 and 4: HBD and Dynamic Stretching Conditions

During visits 3 and 4, participants completed moderate- and severe-intensity cycling following either the HBD or DYN condition. The same condition (i.e., HBD or DYN) was performed before both cycling tests within a given visit. Upon arrival to the laboratory, resting arterial blood pressure was measured, and participants were instrumented for HR, pulmonary gas exchange, and skeletal muscle oxygenation measurements. Participants then completed the same dynamic warm-up described above.

During the HBD condition, all participants successfully completed 3 sets of HBDs: 5 repetitions at 70%, 5 repetitions at 75%, and 5 repetitions at 80% 1RM. The HBD was selected as the conditioning activity because it loads lower-body musculature relevant to cycling, can be individualized relative to each participant’s 1RM, and has been shown to elicit high force, power, velocity, and vastus lateralis activation during resistance exercise [40]. This loading scheme represented a standardized progressive sequence completed by all participants and was selected to provide a moderate-to-heavy, multiple-set conditioning stimulus consistent with prior PAPE literature [9]. The 70–80% 1RM range was selected to provide a moderate-to-heavy submaximal conditioning stimulus while allowing participants to perform each repetition with maximal intended concentric velocity and limiting excessive fatigue before cycling. The same relative HBD loading sequence was used before both the moderate- and severe-intensity cycling tests during the HBD visit. Participants were instructed to perform each repetition with maximal intended velocity during the concentric phase while maintaining proper lifting mechanics. Three minutes of rest were provided between sets. During the DYN condition, participants completed ~8 min of additional dynamic stretching using the same movement series described above rather than HBD exercise. DYN was used as an active control to account for the additional warm-up period and pre-cycling transition without exposure to the HBD stimulus. Following each condition-specific intervention, participants rested for ~8 min before cycling. The ~8-min recovery period was selected because prior PAPE literature suggests that performance enhancement is favored after moderate recovery intervals, particularly ~7–10 min following a conditioning activity [7,9].

The moderate-intensity cycling test was performed first and consisted of 6 min of constant-work-rate cycling at ~90% of the GET. Moderate- and severe-intensity cycling were performed within the same visit to characterize physiological responses to each assigned condition across two exercise intensities while limiting participant burden and between-day variability. The moderate-intensity bout was always completed first because it was fixed-duration and submaximal, whereas the severe-intensity bout was performed to exhaustion and could have influenced subsequent moderate-intensity responses if performed first. After at least 30 min of passive recovery, the same condition-specific intervention (i.e., HBD or DYN) was repeated before the severe-intensity cycling test. The severe-intensity cycling test was performed at 80% of WR_peak_ and continued until exhaustion.

### 2.6. Measurements

#### 2.6.1. Exercise Tolerance

Exercise tolerance (i.e., time-to-exhaustion) was assessed during severe-intensity cycling. Time-to-exhaustion was defined as the time from the onset of the severe-intensity work rate to exercise termination. Tests were terminated due to volitional exhaustion or inability to maintain the required pedal cadence for ≥5 s despite strong verbal encouragement. Severe-intensity time-to-exhaustion was designated as the primary outcome.

#### 2.6.2. Central Hemodynamics

HR was continuously monitored on a beat-by-beat basis from rest through moderate- and severe-intensity exercise using a chest-worn sensor strap (Polar H10, Polar Electro Oy, Kempele, Finland). Arterial blood pressure was measured using an automated upper-arm blood pressure monitor (iHealth Track Connected Blood Pressure Monitor, KN-550BT; iHealth Labs, Inc., Sunnyvale, CA, USA) with a manufacturer-provided wide-range cuff. The cuff was placed on the upper arm according to manufacturer instructions and was appropriate for arm circumferences of 22–42 cm. Arterial blood pressure measurements were obtained at rest and immediately following exercise termination.

#### 2.6.3. Pulmonary Gas Exchange and Ventilation

Resting and exercising pulmonary gas exchange and ventilation were measured breath-by-breath using a calibrated metabolic cart (Quark CPET, Cosmed, Concord, CA, USA). Participants wore a fitted mask connected to the metabolic system for assessment of expired gas concentrations and flow. Pulmonary oxygen uptake (V̇O_2_), carbon dioxide output (VCO_2_), respiratory exchange ratio, and ventilation were measured during maximal incremental, moderate-intensity, and severe-intensity cycling tests. The metabolic system was calibrated before each test according to the manufacturer’s recommendations.

#### 2.6.4. Skeletal Muscle Microvascular Oxygenation

Skeletal muscle oxygenation (S_m_O_2_) of the quadriceps was measured non-invasively and continuously using near-infrared spectroscopy (NIRS; Moxy Muscle Oxygen Monitor; Hutchinson, MN, USA). Before probe placement, the skin over the *vastus lateralis* of each participant’s dominant leg was prepared by shaving excess hair, if necessary, and cleaning the area with an alcohol wipe. The NIRS probe was secured to the skin with adhesive tape and a black elastic bandage to minimize movement artifact and ambient-light interference during cycling; however, the exact distance from anatomical landmarks was not recorded. The probe position was marked to verify placement during the experimental session and to ensure consistent repositioning during the subsequent visit. This approach is consistent with prior work reporting acceptable test-retest reliability for Moxy-derived vastus lateralis S_m_O_2_ during cycling exercise [41,42]. However, between-day reliability for S_m_O_2_ was not directly quantified in the present study. Subcutaneous adipose-tissue thickness at the NIRS site was not measured. However, because each participant served as their own control and probe placement was marked and reproduced across visits, adipose-tissue thickness was expected to remain constant across conditions within each participant. S_m_O_2_ was recorded at rest and throughout moderate- and severe-intensity cycling.

### 2.7. Data Analysis

Pulmonary V̇O_2,_ HR, and S_m_O_2_ data points exceeding 4 standard deviations from the local mean were excluded from analysis [43]. Responses were subsequently time-aligned at exercise onset, linearly interpolated every second (pulmonary V̇O_2_), every 2 s (muscle S_m_O_2_), or averaged into 5 s bins (heart rate) for each participant. Resting V̇O_2,_ HR, and S_m_O_2_ were determined by calculating the average values from 30 s prior to exercise onset to exercise onset, while end-contraction V̇O_2,_ HR, and S_m_O_2_ were determined by calculating the average values from 30 s prior to exercise cessation to exercise cessation.

Kinetics analyses were performed using nonlinear least-squares regression (Marquardt-Levenberg; SigmaPlot 14.5; Systat Software, San Jose, CA, USA). The first 20 s of pulmonary V̇O_2_ data following the onset of exercise (i.e., cardiodynamic phase) were removed from the analysis. Moderate- and severe-intensity V̇O_2_ data were described using mono- and bi-exponential models, respectively:

One-component

$${\dot{V}O}_{2(t)}={\dot{V}O}_{2(rest)}+A_{1}(1-e^{-(t-{TD}_{1}/\tau_{1})})$$

Two-component

$${\dot{V}O}_{2(t)}={\dot{V}O}_{2(rest)}+A_{1}(1-e^{-(t-{TD}_{1}/\tau_{1})})+A_{2}(1-e^{-(t-{TD}_{2}/\tau_{2})})$$

where V̇O_2(t)_ indicates the V̇O_2_ at a given time (t); V̇O_2(rest)_ is the resting value (30 s average) before exercise onset; A_1_ and A_2_ are the amplitudes for the exponential terms; τ_1_ and τ_2_ are the time constants; and TD_1_ and TD_2_ are the independent time delays. Similarly, individual muscle S_m_O_2_ profiles during moderate and severe exercise were described using mono- or bi-exponential models as follows:

One-component

$${S_{t}O}_{2(t)}={S_{t}O}_{2(rest)}-A_{1}(1-e^{-(t-{TD}_{1}/\tau_{1})})$$

Two-component

$${S_{t}O}_{2(t)}={S_{t}O}_{2(rest)}-A_{1}(1-e^{-(t-{TD}_{1}/\tau_{1})})+A_{2}(1-e^{-(t-{TD}_{2}/\tau_{2})})$$

where subscripts t and rest, and parameters A, TD and τ are defined above. Goodness of fit and model selection were determined using three criteria: the coefficient of determination, the sum of squared residuals, and visual inspection. The overall dynamics of the V̇O_2_ and S_m_O_2_ profiles, represented by the mean response time (MRT), were determined as follows [18]:

One-component

$$MRT={TD}_{1}+\tau_{1}$$

Two-component

$$MRT=\left(A_{1}/A_{total}\right)\left({TD}_{1}+\tau_{1}\right)+\left(A_{2}/A_{total}\right)\left({TD}_{2}+\tau_{2}\right)$$

where A_1_ and A_2_, TD_1_ and TD_2_, and τ_1_ and τ_2_ are defined above, and A_total_ is the total change in V̇O_2_(A_1_ + A_2_) when using the two-component model. The MRT analysis for S_m_O_2_ during moderate and severe exercise was constrained to the one-component model, given that the inclusion of the emergent second component underestimates the speed of fall during the onset of muscle contractions [44].

The area under the S_m_O_2_ curve as a function of time (S_m_O_2area_; %·s) was calculated from 30 s of rest to the cessation of exercise during both moderate- and severe-intensity cycling. The S_m_O_2area_ provides an index of overall muscle oxygenation throughout the onset of contractions (i.e., incorporating resting and end-contraction S_m_O_2_, time delays, amplitudes, and time constants of the responses) [45].

Data inclusion for each outcome was determined before final statistical comparisons. After initial filtering and time alignment, pulmonary V̇O_2_, HR, and S_m_O_2_ responses were visually inspected by two investigators for signal quality, artifact, and dropout. For kinetic analyses, individual profiles were retained only when the response demonstrated a coherent rest-to-exercise transition and yielded a physiologically plausible model fit based on the goodness-of-fit criteria described above. Data were excluded from a given outcome when poor signal quality, artifact/dropout, or an unacceptable kinetic model fit precluded valid analysis. These exclusions were determined before condition comparisons and were not based on the direction, magnitude, or statistical significance of the HBD versus DYN response.

### 2.8. Statistical Analysis

All data analyses were performed using commercially available software (SigmaPlot 14.5; Systat Software, San Jose, CA; USA). Comparisons of time-to-exhaustion, pulmonary V̇O_2_ kinetics parameters, quadriceps muscle S_m_O_2_ kinetics parameters_,_ and HR parameters were performed using two-tailed paired Student’s *t*-tests. Arterial blood pressure (systolic, diastolic, and mean arterial pressure (MAP)) was analyzed using two-way repeated-measures analysis of variance (ANOVA). *Post hoc* analyses were conducted using the Student–Newman–Keuls test. Time-to-exhaustion and arterial blood pressure were available for all participants (*n* = 25). HR analyses included *n* = 23 for moderate-intensity cycling and *n* = 24 for severe-intensity cycling due to poor HR signal quality and/or signal dropout. Pulmonary V̇O_2_ kinetics analyses included *n* = 24 for both moderate- and severe-intensity cycling due to poor breath-by-breath signal quality and/or unacceptable kinetic model fit. S_m_O_2_ kinetics analyses included *n* = 18 for moderate-intensity cycling and n = 19 for severe-intensity cycling due to poor NIRS signal quality and/or unacceptable kinetic model fit. Data are presented as means ± SE to describe the precision of the group mean estimates. Significance was accepted at *p* < 0.05. Given the number of condition-related comparisons across pulmonary V̇O_2_ kinetics, muscle S_m_O_2_ kinetics, HR, and arterial blood pressure variables, Benjamini–Hochberg false discovery rate correction was also applied to this family of tests. Unadjusted *p*-values are reported for transparency; however, condition-related findings were interpreted as statistically significant only when the false discovery rate-adjusted *q* value was <0.05. For key paired comparisons, HBD − DYN mean differences, 95% confidence intervals (CIs), and paired-samples Cohen’s *d_z_* effect sizes were calculated to characterize the magnitude and precision of the observed effects. Cohen’s *d_z_* was calculated as the mean paired difference divided by the standard deviation of the paired differences. Positive values indicate greater responses following HBD relative to DYN.

## 3. Results

**Figure 2:**
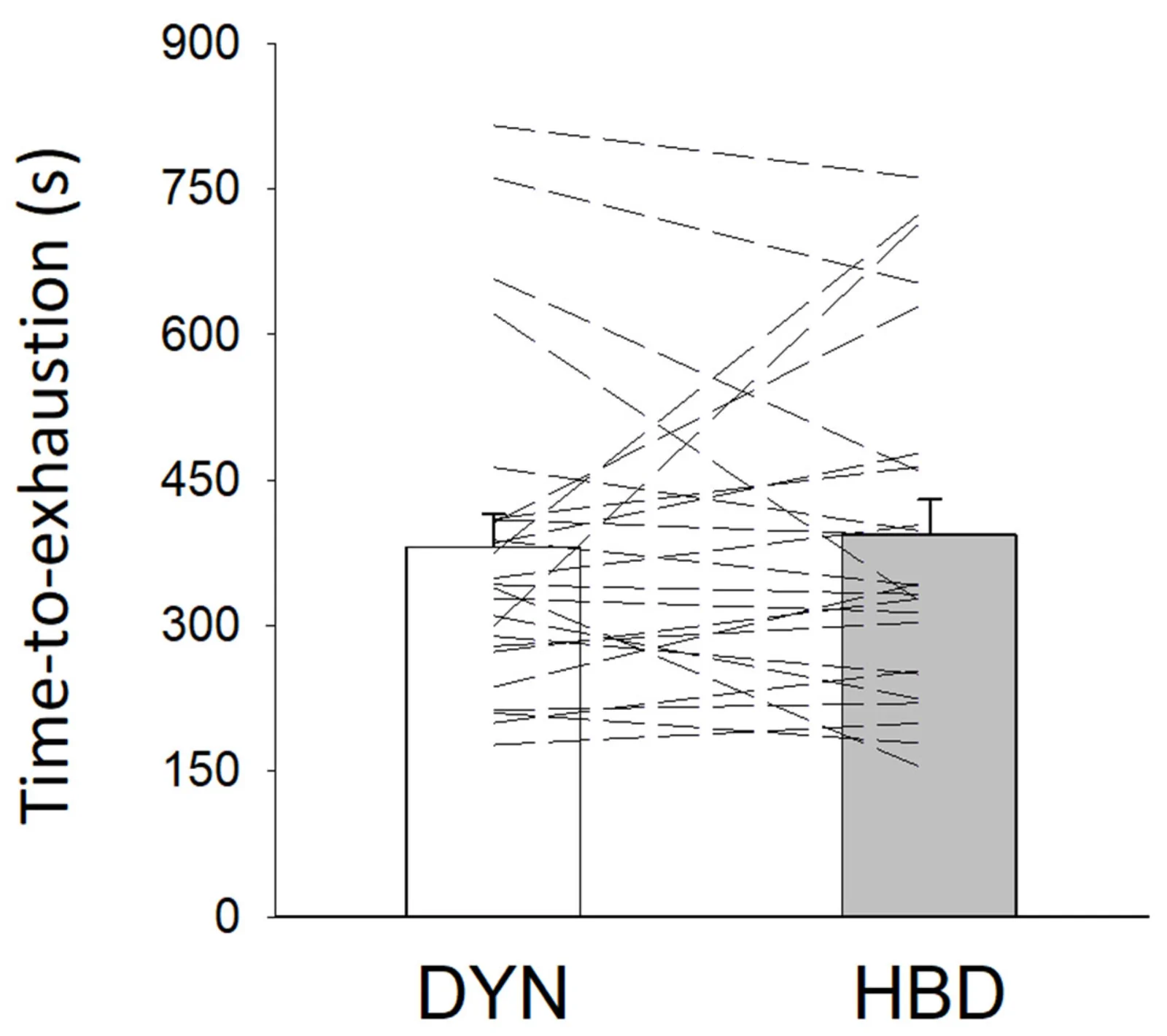
Group mean and individual time-to-exhaustion values during severe-intensity exercise following DYN (*n* = 25) and HBD (*n* = 25). Values are means ± SE. DYN, dynamic stretching; HBD, hex-bar deadlifts.

Participant characteristics, responses to maximal incremental cycling exercise, and maximal hex-bar deadlift results are shown in Table 1. Twenty-five participants, including 15 men and 10 women, completed all experimental visits. Pulmonary V̇O_2peak_ was 2.50 ± 0.15 L/min [32.70 ± 1.60 mL/kg/min], and V̇O_2_ at the gas exchange threshold was 1.71 ± 0.10 L/min [22.34 ± 1.08 mL/kg/min]. WR_peak_ was 206 ± 11 W. There was no significant difference in time-to-exhaustion during severe-intensity cycling between HBD and DYN (HBD: 394 ± 36 s; DYN: 381 ± 34 s; unadjusted *p* = 0.692; Figure 2). The HBD − DYN mean difference was 12.6 s (95% CI: −52.3 to 77.5 s; *d_z_* = 0.08; Table 2).

**Figure 3:**
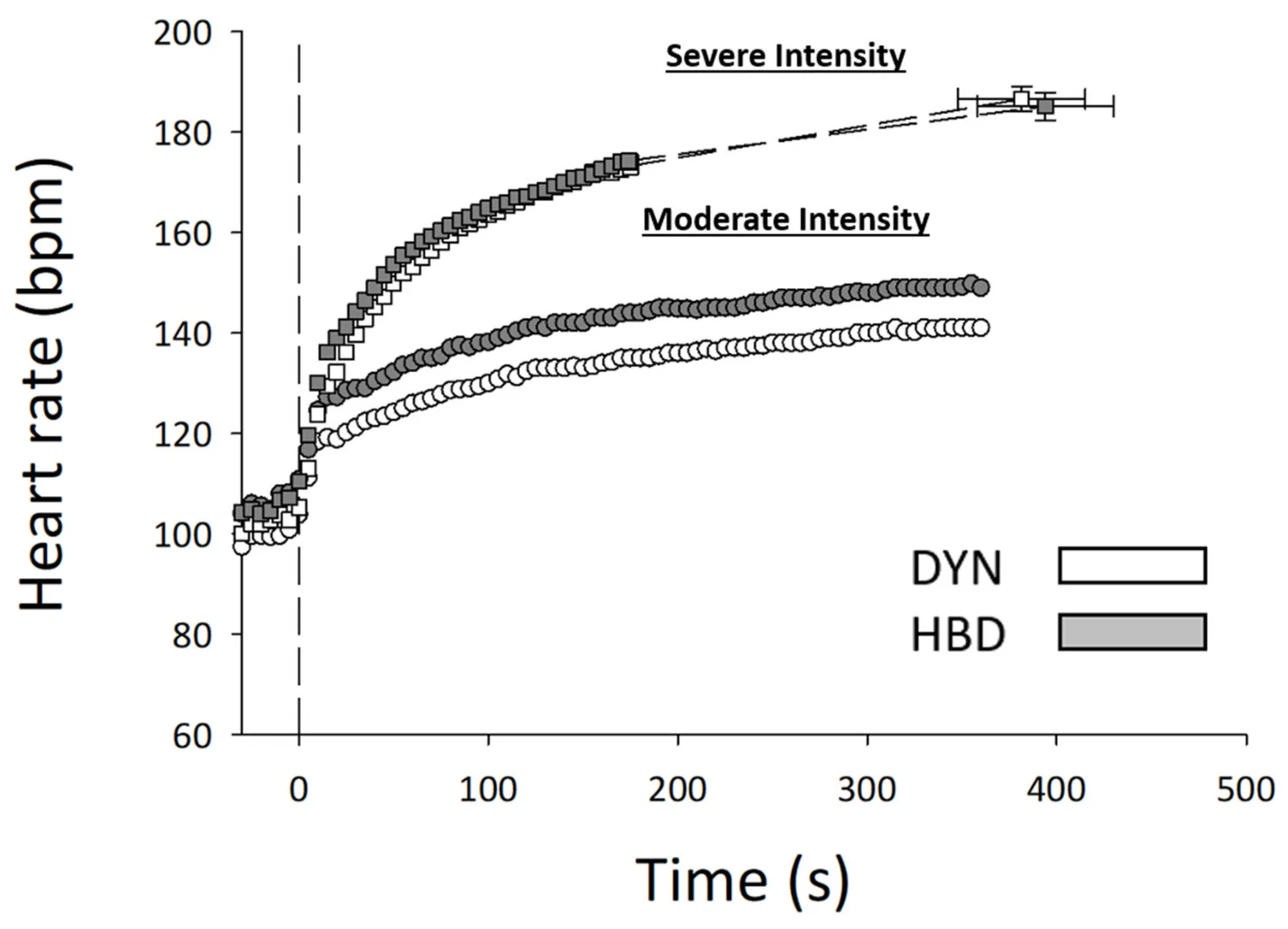
Group mean heart rate from rest to moderate (DYN, *n* = 23; HBD, *n* = 23) and severe (DYN, *n* = 24; HBD, *n* = 24) exercise following DYN and HBD. Values are means ± SE. Error bars omitted for clarity (except for maximal values during severe intensity exercise). Exercise started at *time 0*. DYN, dynamic stretching; HBD, hex-bar deadlift.

Group mean HR responses from rest to moderate- and severe-intensity cycling are shown in Figure 3. Resting HR before moderate-intensity cycling displayed a nominal unadjusted difference between conditions (HBD: 106 ± 3 bpm; DYN: 99 ± 4 bpm; unadjusted *p* = 0.032); however, this comparison did not remain significant after false discovery rate correction (*q* = 0.809). End-contraction HR was not different between conditions during moderate-intensity cycling (HBD: 149 ± 4 bpm; DYN: 141 ± 7 bpm; *p* = 0.254). During severe-intensity cycling, there were no significant differences in resting HR (HBD: 105 ± 3 bpm; DYN: 102 ± 3 bpm; *p* = 0.163) or end-contraction HR (HBD: 185 ± 3 bpm; DYN: 187 ± 2 bpm; *p* = 0.329) between conditions.

Arterial blood pressure responses are shown in Table 3. Systolic blood pressure and MAP increased from pre- to post-exercise during both moderate- and severe-intensity cycling (main effect of time, *p* < 0.001 for all), with no significant condition or condition × time interaction effects (*p* > 0.05 for all). Diastolic blood pressure did not change from pre- to post-exercise and was not different between conditions during either exercise intensity (*p* > 0.05 for all).

**Figure 4:**
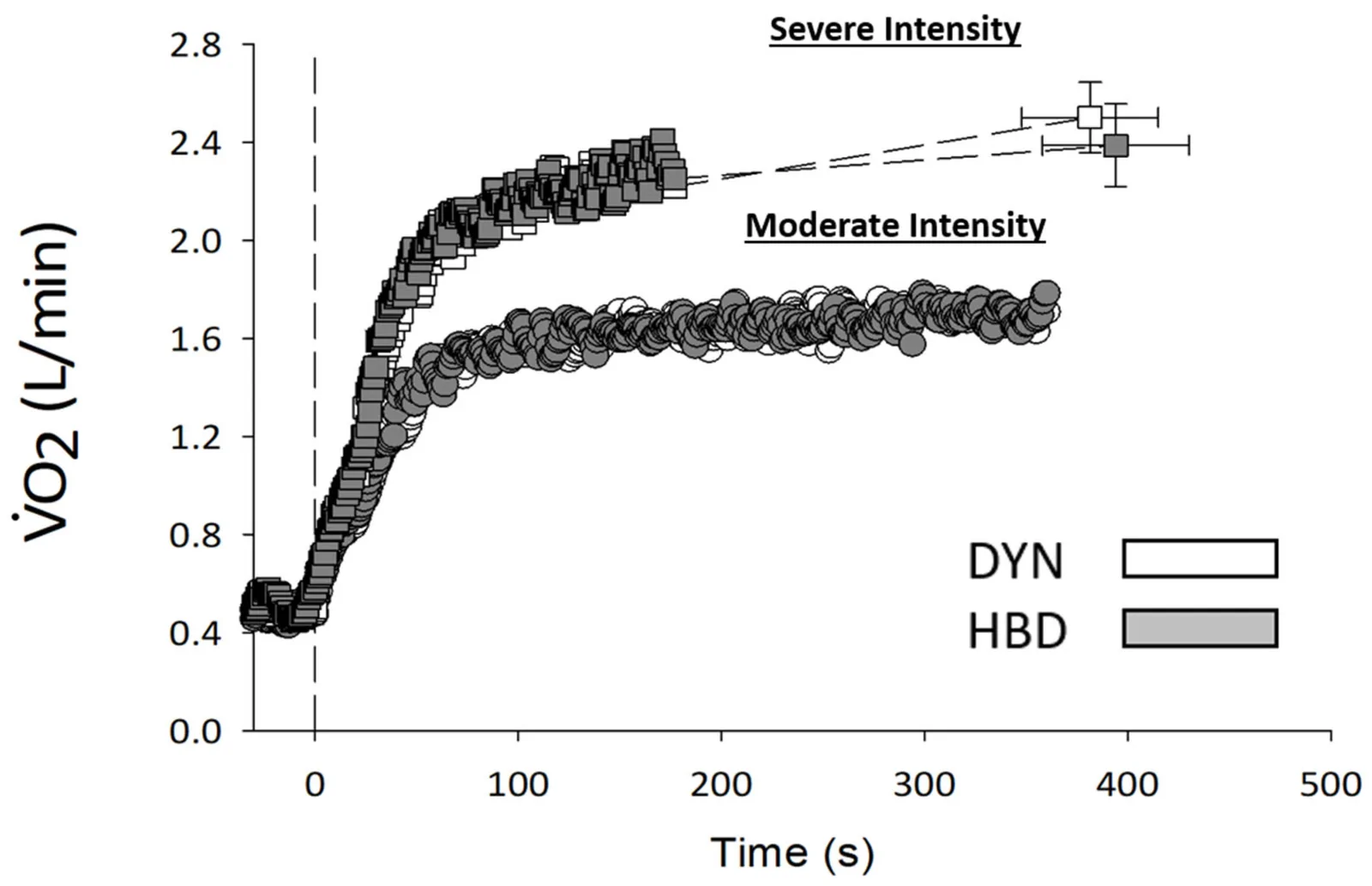
Group mean pulmonary oxygen uptake (V̇O_2_) from rest to moderate (DYN, *n* = 24; HBD, *n* = 24) and severe (DYN, *n* = 24; HBD, *n* = 24) exercise following DYN and HBD. Values are means ± SE. Error bars omitted for clarity (except for maximal values during severe intensity exercise). Exercise started at *time* 0. DYN, dynamic stretching; HBD, hex-bar deadlift.

Pulmonary V̇O_2_ responses from rest to moderate- and severe-intensity cycling following HBD and DYN are shown in Figure 4. Resting and end-contraction V̇O_2_ values and kinetics parameters are presented in Table 4. Resting and end-contraction V̇O_2_ were not different between conditions during moderate- or severe-intensity cycling (*p* > 0.05 for all). There were no significant differences in V̇O_2_ kinetics during moderate-intensity cycling between conditions (*p* > 0.05 for all parameters). During severe-intensity cycling, no V̇O_2_ kinetic parameter remained significantly different between conditions after false discovery rate correction. The second-component time delay displayed a nominal unadjusted difference between HBD and DYN (unadjusted *p* = 0.043; *q* = 0.809), but this response was not interpreted as statistically significant. For V̇O_2_ MRT, the HBD − DYN mean differences were −2.34 s during moderate-intensity cycling (95% CI: −8.06 to 3.37 s; *d_z_* = −0.17) and −15.70 s during severe-intensity cycling (95% CI: −37.20 to 5.80 s; *d_z_* = −0.31; Table 2).

**Figure 5:**
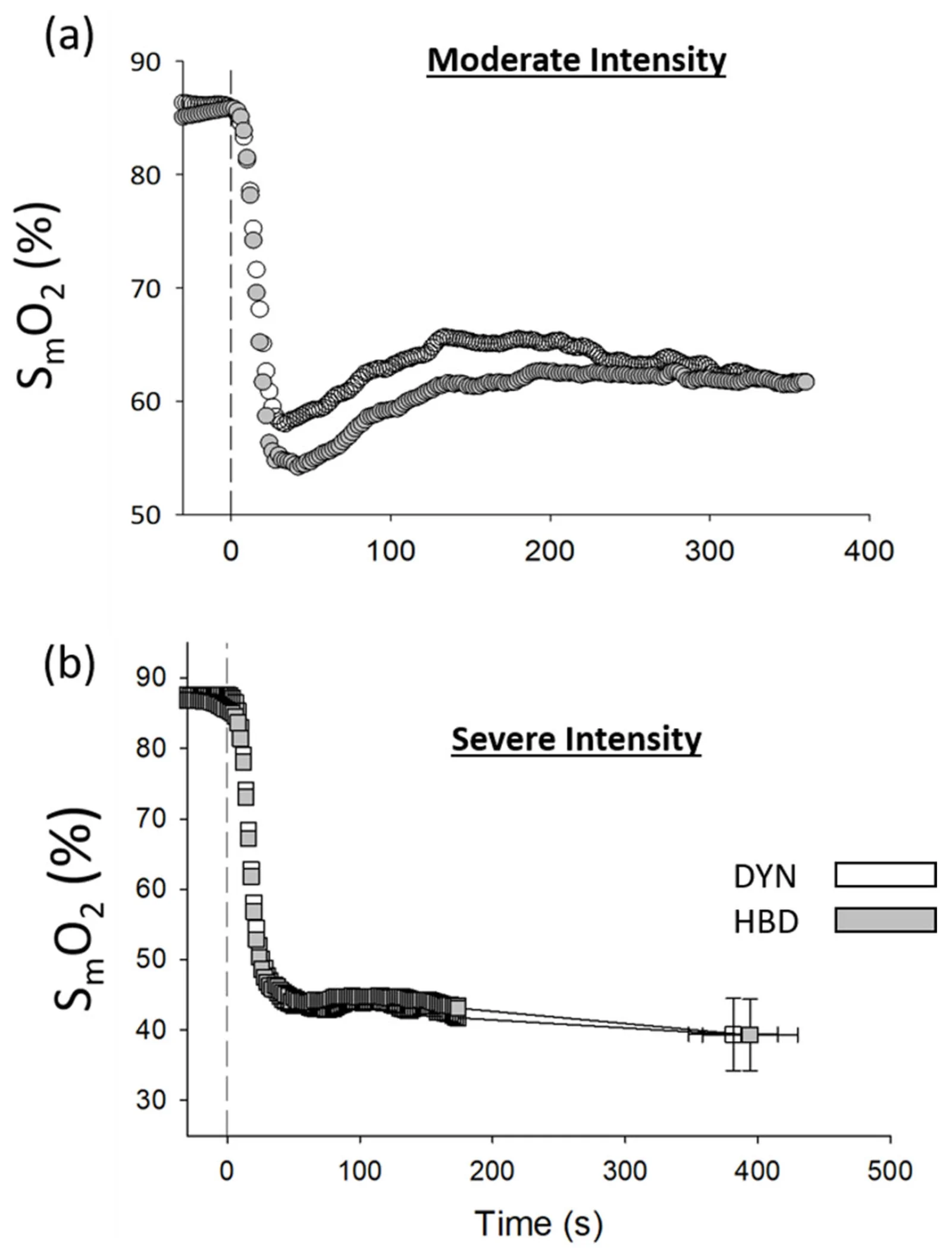
Group mean leg skeletal muscle oxygen saturation (S_m_O_2_) from rest to moderate (DYN, *n* = 18; HBD, *n* = 18) and severe (DYN, *n* = 19; HBD, *n* = 19) exercise following DYN and HBD. (**a**) Moderate intensity, (**b**) Severe intensity.

Quadriceps muscle S_m_O_2_ responses from rest to moderate- and severe-intensity cycling following HBD and DYN are shown in Figure 5. Resting and end-contraction S_m_O_2_ values and the kinetic parameters are presented in Table 5. Resting and end-contraction S_m_O_2_ were not different between conditions during moderate- or severe-intensity cycling (*p* > 0.05 for all). Similarly, there were no significant differences in muscle S_m_O_2_ kinetics between conditions during either exercise intensity (*p* > 0.05 for all parameters). S_m_O_2area_ was not different between HBD and DYN during moderate-intensity cycling (HBD: 25,036 ± 1650%·s; DYN: 25,283 ± 1771%·s; unadjusted *p* = 0.800; mean difference: −247%·s, 95% CI: −2254 to 1760%·s; *d_z_* = −0.06) or severe-intensity cycling (HBD: 21,802 ± 3102%·s; DYN: 19,610 ± 2526%·s; unadjusted *p* = 0.255; mean difference: 2192%·s, 95% CI: −1702 to 6086%·s; *d_z_* = 0.25; Table 2). For S_m_O_2_ MRT, the HBD − DYN mean differences were −0.24 s during moderate-intensity cycling (95% CI: −6.12 to 5.65 s; *d_z_* = −0.02) and −7.48 s during severe-intensity cycling (95% CI: −19.42 to 4.46 s; *d_z_* = −0.30; Table 2).

## 4. Discussion

The present study examined the effects of acute HBD exercise, used as a conditioning activity intended to induce PAPE, on subsequent cycling performance and skeletal muscle microvascular oxygenation in healthy young adults. Contrary to our hypothesis, the present data do not provide clear evidence that HBD improved severe-intensity cycling tolerance or enhanced vastus lateralis muscle oxygenation during subsequent cycling. Specifically, HBD did not improve time to exhaustion during severe-intensity cycling, increase resting or end-contraction S_m_O_2_, alter S_m_O_2_ kinetics, or improve the overall S_m_O_2_ response during moderate- or severe-intensity cycling. After correction for multiple comparisons, no condition-related differences in pulmonary V̇O_2_ kinetics, S_m_O_2_ kinetics, HR, or arterial blood pressure remained statistically significant. In addition, the paired mean difference for time-to-exhaustion was small and imprecise, and the 95% CIs for S_m_O_2area_ and MRT crossed zero. Taken together, these findings do not support a consistent meaningful effect of HBD exercise on skeletal muscle O_2_ delivery-utilization matching or severe-intensity cycling tolerance under the conditions evaluated herein.

The lack of improvement in severe-intensity cycling performance is important given the growing interest in applying PAPE-type strategies beyond explosive or ballistic tasks. PAPE has been most consistently examined in jump, sprint, throw, and other power-dependent outcomes, where the ability to rapidly produce force is central to performance [7,9,10]. In contrast, cycling exercise tolerance is not governed solely by peak muscle force production or neuromuscular power. Rather, severe-intensity cycling reflects the integrated regulation of central and peripheral O_2_ transport, muscle oxidative metabolism, metabolite accumulation, neuromuscular fatigue, and perceptual strain [5,15,16,17]. Therefore, a conditioning activity that has the potential to improve force-generating capacity does not necessarily translate to improved endurance exercise performance, particularly if residual fatigue or metabolic strain offsets any potentiating effect.

The present results align with the broader view that the ergogenic effect of PAPE on endurance exercise is inconsistent. Previous work has reported improved cycling performance following prior heavy resistance exercise or resistance-based warm-up strategies. For example, Silva et al. reported improved 20 km cycling time-trial performance following prior heavy strength exercise [33], while Chorley and Lamb reported improved 4 km cycling time-trial performance when heavy-resistance conditioning contractions were included in the cycling warm-up [35]. Similarly, de Poli et al. reported improved cycling performance following drop jumps, which was attributed to post-activation potentiation and increased anaerobic capacity [34]. However, those studies differ from the present investigation in participant training status, conditioning activity, performance task, and recovery structure. The present study used a fixed-load HBD stimulus followed by constant-work-rate cycling to the limit of tolerance in healthy young adults with some resistance training experience, but who were not trained cyclists or formal athletes. Thus, the current findings do not rule out the possibility that PAPE may benefit specific cycling performances under different circumstances. Rather, they indicate that HBD performed 8 min before severe-intensity cycling does not provide a consistent ergogenic advantage in this population. The effect size and confidence interval data further support a cautious interpretation of the performance outcome. The HBD − DYN mean difference for time-to-exhaustion was 12.6 s, with a 95% CI ranging from −52.3 to 77.5 s and a small paired effect size (*d_z_* = 0.08). Thus, although the mean response favored HBD numerically, the magnitude of this difference was small relative to the variability of the response. The confidence interval also includes values consistent with both a modest decrease and a modest improvement in performance. Therefore, the present findings should not be interpreted as definitive evidence that HBD has no effect on cycling tolerance, but rather that this protocol did not provide clear or consistent evidence of an ergogenic benefit.

A likely explanation is that the HBD stimulus did not produce a favorable potentiation-fatigue balance for the subsequent cycling task. PAPE is not simply the result of “activation” carrying over into performance, but rather the final response reflects the interaction between potentiating mechanisms and fatigue-related processes [7,27,28]. Acute heavy muscle contractions may increase muscle temperature, alter muscle water content, enhance neural activation, and improve force-generating capacity [7,24]. On the other hand, the same stimulus can increase metabolic strain, disturb the intramuscular environment, and elevate the energetic cost of subsequent exercise [29,30]. In the present study, participants performed 15 total HBD repetitions across 70–80% 1RM with maximal intended concentric velocity. It is also important to note that the moderate-intensity bout was always completed before the severe-intensity bout, and HBD was performed before both cycling bouts within a given visit; therefore, the severe-intensity trial reflects a repeated-intervention protocol rather than a single isolated PAPE bout. Although at least 30 min of passive recovery separated the cycling tests, residual neuromuscular and/or metabolic fatigue from the first HBD bout, the moderate-intensity cycling bout, or both could have attenuated any potentiating effect of the second HBD stimulus. Thus, any potential beneficial effect on neuromuscular readiness, muscle temperature, or local perfusion was either too small to detect, not specific to the cycling task, or counterbalanced by residual fatigue or metabolic strain.

The task-specific nature of the HBD stimulus should also be considered. The HBD is a hip- and knee-extension-dominant movement characterized by relatively high force production and brief contraction duration. In contrast, severe-intensity cycling requires repeated rhythmic contractions, sustained ATP turnover, and tolerance of progressive metabolic and neuromuscular fatigue. The lack of improvement in time-to-exhaustion may therefore reflect limited transfer between the conditioning contraction and the subsequent performance task. This is especially relevant because task specificity and individual characteristics, including strength level and training status, modulate the PAPE response [9,10]. It is possible that stronger or more highly trained individuals would display a more favorable potentiation-fatigue profile following HBD. Conversely, in healthy young adults without formal athletic training, the same protocol may have imposed enough fatigue to neutralize any potentiating benefit.

The central mechanistic question was whether HBD would improve skeletal muscle oxygenation during subsequent cycling; however, the present data do not support this effect. Resting and end-contraction S_m_O_2_ were not different between HBD and DYN during either moderate- or severe-intensity cycling. Similarly, S_m_O_2_ kinetics and S_m_O_2area_ were unchanged. These findings do not support the hypothesis that HBD improves quadriceps O_2_ delivery relative to O_2_ utilization during subsequent cycling. Given that NIRS-derived S_m_O_2_ does not measure blood flow directly, these data should not be interpreted as proof that limb perfusion was unchanged. Rather, S_m_O_2_ reflects the local balance between microvascular O_2_ delivery and muscle O_2_ utilization within the interrogated tissue volume [6,31]. Within this context, the absence of condition differences across several S_m_O_2_ indices suggests that the net local O_2_ delivery–utilization balance was not meaningfully altered by HBD.

This result is physiologically relevant because prior muscle contractions can, under some conditions, alter the microvascular O_2_ response during subsequent contractions. Behnke et al. demonstrated that prior contractions can modify muscle microvascular partial pressure of oxygen dynamics at the onset of subsequent contractions [23], consistent with the concept that the contractile history of skeletal muscle may influence local O_2_ delivery-utilization matching. In the present study, however, HBD did not increase S_m_O_2_ at rest, slow the fall in S_m_O_2_ at exercise onset, elevate end-contraction S_m_O_2_, or increase S_m_O_2area_. One possibility is that any local hyperemia or vasodilatory effect induced by HBD had largely dissipated during the ~8-min recovery period. Another possibility is that cycling itself rapidly became the dominant regulator of local O_2_ delivery and utilization, thereby minimizing any residual effect of the preceding HBD bout. Finally, an increase in O_2_ delivery after HBD may have been matched by a proportional increase in O_2_ utilization, resulting in no detectable change in S_m_O_2_. Within this context, the absence of condition differences across several S_m_O_2_ indices suggests that the net local O_2_ delivery-utilization balance was not clearly altered by HBD. However, the confidence interval for severe-intensity S_m_O_2area_ was relatively wide, indicating that a modest effect in either direction cannot be excluded. Therefore, these findings are best interpreted as showing no clear evidence that HBD enhanced vastus lateralis oxygenation during subsequent cycling, rather than definitive evidence that HBD has no effect on peripheral O_2_ transport.

The pulmonary V̇O_2_ data also do not support a clear priming effect of HBD. Prior heavy exercise can accelerate pulmonary V̇O_2_ kinetics, reduce the V̇O_2_ slow component, and improve severe-intensity exercise tolerance under certain conditions [18,20,21,22]. These effects are generally interpreted to reflect improved adjustment of O_2_ delivery and/or O_2_ utilization during the transition to high-intensity exercise [5,26]. In the present study, resting and end-contraction V̇O_2_ were not different between conditions, and no V̇O_2_ kinetic parameter remained significantly different after false discovery rate correction. Moreover, the HBD − DYN mean differences for V̇O_2_ MRT were −2.34 s during moderate-intensity cycling and −15.70 s during severe-intensity cycling, with both 95% CIs crossing zero. Therefore, the present data do not provide clear evidence that HBD accelerated the overall pulmonary V̇O_2_ response during subsequent cycling.

The central hemodynamic responses further support the conclusion that HBD did not clearly improve the integrated O_2_ transport response to cycling. Resting HR before moderate-intensity cycling displayed a nominal unadjusted increase following HBD compared with DYN, which may reflect residual sympathetic activation, increased arousal, or incomplete autonomic recovery after the conditioning activity. However, this comparison did not remain significant after correction for multiple comparisons and was not present at end-contraction. Moreover, resting and end-contraction HR were not different between conditions during severe-intensity cycling, and arterial blood pressure responses were not different between HBD and DYN. Thus, the HBD protocol did not alter central hemodynamic responses in a manner that translated to changes in pulmonary V̇O_2_, skeletal muscle S_m_O_2_, or severe-intensity exercise tolerance.

From an applied standpoint, the present data do not support HBD as a general warm-up strategy to improve severe-intensity cycling tolerance in healthy young adults. This does not mean that resistance-based conditioning activities should be dismissed entirely for endurance or cycling exercise contexts. Rather, the response likely depends on the athlete, the conditioning stimulus, the recovery period, and the performance task. For applied settings, a fixed HBD prescription and fixed recovery period may not be sufficient. Individualized recovery durations, velocity-based monitoring of the conditioning activity, and task-specific priming strategies may be necessary to identify responders and avoid carrying fatigue into the subsequent performance.

Several limitations should be acknowledged. First, classical PAP was not directly assessed. We did not measure electrically evoked twitch force before and after HBD; therefore, the present study appropriately evaluates PAPE rather than PAP [7,8]. Second, we did not measure muscle activation, motor unit recruitment, or bar velocity during the HBD stimulus. Participants were instructed to lift with maximal intended concentric velocity, but actual concentric velocity and power output were not quantified. Third, we did not directly assess muscle temperature, blood lactate, muscle metabolites, or perceived exertion. These measurements would have provided insights into whether the HBD stimulus increased readiness, residual fatigue, or metabolic strain before cycling. Fourth, NIRS-derived S_m_O_2_ reflects local O_2_ delivery-utilization balance and does not represent blood flow per se. Thus, the present study cannot determine whether HBD altered femoral artery blood flow, vascular conductance, microvascular hematocrit, or O_2_ extraction independently. In addition, the reduced sample size for S_m_O_2_ kinetics due to poor signal quality and/or unacceptable model fit may have limited statistical power to detect condition-related differences in skeletal muscle oxygenation. Another limitation is that test-retest reliability was not established for the severe-intensity constant-work-rate time-to-exhaustion test used herein. This is important because time-to-exhaustion tests can exhibit considerable within-subject variability and may be influenced by familiarization or learning effects [46,47]. Although the randomized crossover design helped minimize systematic order effects, the absence of a separate familiarization or reliability trial limits our ability to determine whether the null effect of HBD on time-to-exhaustion reflects a true absence of effect or variability inherent to the test. Therefore, the time-to-exhaustion outcome should be interpreted cautiously. Additionally, although the paired effect sizes for the key outcomes were generally small, several 95% CIs were wide; therefore, the present findings should be interpreted as exploratory and should not be taken as definitive evidence that HBD has no physiological or performance effect. Moreover, since the condition-specific intervention was performed before both cycling bouts, the severe-intensity trial should be interpreted as the response to a repeated-intervention protocol rather than a single isolated PAPE bout. Although at least 30 min of passive recovery separated the cycling tests, we cannot exclude the possibility that the first intervention, moderate-intensity cycling bout, or their combination influenced responses to the second intervention and subsequent severe-intensity test. In addition, the study population included healthy young adults with some resistance training experience, but excluded formal athletes and individuals following supervised exercise programs. These criteria were useful for limiting the influence of high training status, but they also limit generalizability to trained cyclists, strength-power athletes, or other competitive athletes. Menstrual-cycle phase and hormonal-contraceptive use were also not recorded or controlled in female participants; therefore, hormonal variability may have influenced cardiovascular, metabolic, skeletal muscle oxygenation, or performance responses. Accordingly, the present findings should be interpreted as pooled responses from healthy young adults and not as sex-specific responses to HBD-based PAPE.

Future work should examine whether the response to HBD differs across training status, strength level, sex, and cycling specialization. It may also be useful to individualize the recovery period following HBD, as the optimal time course of PAPE likely varies across individuals. Combining HBD with measurements of bar velocity, electromyography, muscle temperature, blood lactate, RPE, and limb blood flow would help separate potentiating mechanisms from fatigue-related processes. Finally, future studies may consider shorter cycling performance tasks, such as a 1-km or 4-km time trial, where neuromuscular power and anaerobic capacity may contribute more strongly to performance than during severe-intensity time-to-exhaustion cycling.

## 5. Conclusions

In conclusion, acute HBD exercise performed ~8 min before cycling did not provide clear evidence of improved severe-intensity exercise tolerance or enhanced quadriceps S_m_O_2_ during moderate- or severe-intensity cycling. No condition-related differences in HR, arterial blood pressure, pulmonary V̇O_2_, or skeletal muscle S_m_O_2_ remained significant after correction for multiple comparisons. Therefore, the physiological effects of HBD-based PAPE do not appear to extend meaningfully to skeletal muscle O_2_ delivery-utilization matching or severe-intensity cycling tolerance under the conditions evaluated herein.

## Figures and Tables

**Table 1:** Subject characteristics, ramp-incremental exercise responses, and maximal hex-bar deadlift results.

Characteristic	Value
**Demographic**	
n (male/female)	25 (15/10)
Age, yr	22 ± 1
Height, cm	169 ± 2
Weight, kg	78.3 ± 4.9
Body mass index, kg/m^2^	27.1 ± 1.1
**Incremental Exercise**	
Peak work rate, W	206 ± 11
Time to exhaustion, s	674 ± 37
V̇**O_2peak_**	
L/min	2.50 ± 0.15
mL/kg/min	32.70 ± 1.60
V̇**O_2_ at gas exchange threshold**	
L/min	1.71 ± 0.10
mL/kg/min	22.34 ± 1.08
**HR, bpm**	
Rest	104 ± 4
Peak exercise	193 ± 2
**Maximal Hex-Bar Deadlift**	
1RM, kg	124 ± 12
Relative strength, kg 1RM/kg body mass	1.59 ± 0.12
**Submaximal Hex-Bar Deadlift**	
70% 1RM, kg	87 ± 8
75% 1RM, kg	93 ± 9
80% 1RM, kg	99 ± 10

Values are means ± SE. V̇O_2peak_, peak pulmonary oxygen uptake; V̇O_2_, pulmonary oxygen uptake; HR, heart rate; 1RM, one-repetition maximum.

**Table 2:** Paired mean differences, 95% confidence intervals, and effect sizes for time-to-exhaustion and key physiological outcomes.

Outcome	Intensity	*n*	Mean Difference, HBD − DYN	95% CI	Cohen’s *d*_z_	Unadjusted *p*
Time-to-exhaustion	Severe	25	12.6 s	−52.3 to 77.5 s	0.08	0.692
S_m_O_2area_	Moderate	20	−247%·s	−2254 to 1760%·s	−0.06	0.800
S_m_O_2area_	Severe	22	2192%·s	−1702 to 6086%·s	0.25	0.255
V̇O_2_ MRT	Moderate	24	−2.34 s	−8.06 to 3.37 s	−0.17	0.405
V̇O_2_ MRT	Severe	24	−15.70 s	−37.20 to 5.80 s	−0.31	0.144
S_m_O_2_ MRT	Moderate	18	−0.24 s	−6.12 to 5.65 s	−0.02	0.933
S_m_O_2_ MRT	Severe	19	−7.48 s	−19.42 to 4.46 s	−0.30	0.204

Values are HBD − DYN paired mean differences. Positive values indicate greater responses following HBD relative to DYN. CI, confidence interval; DYN, dynamic stretching; HBD, hex-bar deadlift; MRT, mean response time; S_m_O_2area_, area under the S_m_O_2_ curve; V̇O_2_, pulmonary oxygen uptake.

**Table 3:** Systolic, diastolic, and mean arterial pressure (MAP) at rest and during moderate- and severe-intensity exercise following DYN (dynamic stretching) and hex-bar deadlifts (HBD).

	Moderate Intensity		Severe Intensity	
	DYN	HBD	DYN	HBD
**Systolic**				
Rest, mmHg	123 ± 1	121 ± 2	120 ± 2	121 ± 2
Exercise, mmHg	150 ± 3 #	146 ± 3 #	165 ± 4 #	161 ± 4 #
**Diastolic**				
Rest, mmHg	81 ± 1	82 ± 1	81 ± 1	81 ± 1
Exercise, mmHg	83 ± 1	84 ± 1	81 ± 2	80 ± 1
**MAP**				
Rest, mmHg	95 ± 1	95 ± 1	94 ± 1	94 ± 1
Exercise, mmHg	105 ± 1 #	104 ± 2 #	109 ± 2 #	107 ± 2 #

Values are means ± SE. Moderate intensity: DYN, *n* = 25; HBD, *n* = 25. Severe intensity: DYN, *n* = 25; HBD, *n* = 25. **#** Significantly different from rest.

**Table 4:** Pulmonary oxygen uptake (V̇O_2_) kinetics from rest to moderate- and severe-intensity exercise following dynamic stretching (DYN) and hex-bar deadlifts (HBD).

	Moderate Intensity		Severe Intensity	
	DYN	HBD	DYN	HBD
V̇O_2rest_, L/min	0.49 ± 0.03	0.50 ± 0.03	0.50 ± 0.04	0.51 ± 0.04
τ_1_, s	33.7 ± 3.0	31.9 ± 2.2	22.1 ± 2.7	24.8 ± 3.4
A_1_, L/min	1.20 ± 0.09	1.18 ± 0.10	1.65 ± 0.09	1.73 ± 0.13
TD_1_, s	7.7 ± 1.6	7.1 ± 1.4	11.8 ± 1.3	11.6 ± 1.5
τ_2_, s	-	-	151.5 ± 22.2	107.4 ± 17.5
A_2_, L/min	-	-	0.52 ± 0.06	0.48 ± 0.05
TD_2_, s	-	-	119.5 ± 12.8	93.6 ± 9.7 ^†^
MRT, s	41.3 ± 2.5	39.0 ± 1.7	88.3 ± 7.8	72.6 ± 6.3
V̇O_2end-contraction_, L/min	1.69 ± 0.11	1.69 ± 0.11	2.50 ± 0.14	2.39 ± 0.17

Values are means ± SE. Moderate intensity: DYN, *n* = 24; HBD, *n* = 24. Severe intensity: DYN, *n* = 24; HBD, *n* = 24. All moderate-intensity profiles were analyzed with the one-component model, while all severe-intensity profiles were analyzed with the two-component model. V̇O_2rest_, resting V̇O_2_; τ_1_, phase II time constant; A_1_, phase II amplitude; TD_1_, phase II time delay; τ_2_, slow component time constant; A_2_, slow component amplitude; TD_2_, slow component time delay; MRT, mean response time; V̇O_2end-contraction_, end-contraction V̇O_2_. ^†^ Nominal unadjusted *p* < 0.05 versus DYN; not significant after false discovery rate correction.

**Table 5:** Leg skeletal muscle oxygen saturation (S_m_O_2_) from rest to moderate- and severe-intensity exercise following dynamic stretching (DYN) and hex-bar deadlifts (HBD).

	Moderate Intensity		Severe Intensity	
	DYN	HBD	DYN	HBD
S_m_O_2rest_, %	86.0 ± 2.1	85.5 ± 1.9	87.2 ± 1.7	86.2 ± 1.6
τ_1_, s	11.8 ± 1.7	11.4 ± 1.6	28.2 ± 10.3	22.2 ± 7.5
A_1_, %	34.6 ± 4.2	31.6 ± 4.2	53.1 ± 4.0	48.8 ± 4.2
TD_1_, s	10.1 ± 1.2	10.2 ± 0.6	10.0 ± 1.6	8.4 ± 0.9
τ_2_, s	65.0 ± 12.0	77.8 ± 26.8	50.0 ± 10.7	36.0 ± 17.4
A_2_, %	13.1 ± 8.3	12.8 ± 2.2	19.9 ± 6.4	13.7 ± 2.6
TD_2_, s	33.6 ± 4.6	50.2 ± 9.8	51.7 ± 15.0	43.2 ± 9.0
MRT, s	21.9 ± 2.8	21.7 ± 1.7	42.8 ± 10.3	35.4 ± 7.0
S_m_O_2end-contraction_, %	61.1 ± 4.9	62.5 ± 4.6	39.4 ± 5.1	39.3 ± 5.1
S_m_O_2area_, %·s	25,282 ± 1771	25,036 ± 1650	19,610 ± 2526	21,802 ± 3102

Values are means ± SE. Moderate exercise: DYN, *n* = 18; HBD, *n* = 18. Severe exercise: DYN, *n* = 19; HBD, *n* = 19. Most moderate-intensity profiles were analyzed with the two-component model (DYN = 16/18, HBD = 15/18). Most severe-intensity profiles were analyzed with the one-component model (DYN = 15/19; HBD = 13/19). S_m_O_2rest_, resting S_m_O_2_; τ_1_, time constant for the first component; A_1_, amplitude of the first component; TD_1_, time delay of the first component; τ_2_, time constant for the second component; A_2_, amplitude of the second component; TD_2_, time delay of second component; MRT, mean response time; S_m_O_2end-contraction_, end-contraction S_m_O_2_; S_m_O_2area_, area under the S_m_O_2_ curve.

## Data Availability

The original contributions presented in this study are included in the article. Further inquiries can be directed to the corresponding author(s).

## Abbreviations

The following abbreviations are used in this manuscript:

1RM	One-repetition maximum
A_1_	Amplitude of the first component
A_2_	Amplitude of the second component
ANOVA	Analysis of variance
BMI	Body mass index
DYN	Dynamic stretching
GET	Gas exchange threshold
HBD	Hex-bar deadlift
HR	Heart rate
MAP	Mean arterial pressure
MRT	Mean response time
NIRS	Near-infrared spectroscopy
O_2_	Oxygen
PAP	Post-activation potentiation
PAPE	Post-activation performance enhancement
SE	Standard error
S_m_O_2_	Skeletal muscle oxygen saturation
S_m_O_2area_	Area under the SmO2 curve
S_m_O_2end-contraction_	End-contraction skeletal muscle oxygen saturation
S_m_O_2rest_	Resting skeletal muscle oxygen saturation
TD	Time delay
TD_1_	Time delay of the first component
TD_2_	Time delay of the second component
V̇CO_2_	Carbon dioxide output
V̇O_2_	Pulmonary oxygen uptake
V̇O_2end-contraction_	End-contraction pulmonary oxygen uptake
V̇O_2peak_	Peak pulmonary oxygen uptake
V̇O_2rest_	Resting pulmonary oxygen uptake
WR_peak_	Peak work rate
τ	Time constant
τ_1_	Time constant for the first component
τ_2_	Time constant for the second component
